# Reoperation and Complication Rates Following Percutaneous Bone Graft and Headless Screw Fixation for Scaphoid Non-union: A 10-Year, Single-Centre Experience

**DOI:** 10.7759/cureus.117216

**Published:** 2026-09-30

**Authors:** Aiman Awad, Anthony Humphrey

**Affiliations:** 1 Trauma and Orthopaedics, County Durham and Darlington NHS Foundation Trust, Darlington, GBR

**Keywords:** minimally invasive scaphoid fixation, percutaneous scaphoid bone graft, percutaneous scaphoid fixation, scaphoid fracture non union, treatment of scaphoid nonunion

## Abstract

Background and objective

Percutaneous fixation techniques have been recommended for certain scaphoid non-unions as they limit soft tissue disruption and may help preserve scaphoid vascularity. Adding percutaneous bone grafting aims to improve biological healing while keeping the approach minimally invasive. However, the evidence on outcomes is still limited. This study evaluated the radiographic and clinical outcomes of percutaneous bone grafting combined with screw fixation for the treatment of scaphoid non-union.

Methods

A retrospective review was conducted involving all patients who underwent percutaneous iliac crest bone grafting and headless compression screw fixation for scaphoid non-union by a single surgeon at a single institution between 2010 and 2020. Demographic data, fracture characteristics, union status, complications, and reoperations were documented.

Results

The study included 45 consecutive patients with accessible clinical records. The mean age was 26.3 ± 11.2 years (range: 16-63 years), and 41 (91%) were male. The scaphoid waist was involved in most non-unions (41/45, 91%), while four affected the proximal pole. Patients were followed for a mean of 591.9 ± 508.1 days (median: 502 days; interquartile range (IQR): 216-653.5 days). Radiographic union was observed in 15 patients, with a mean time to first documented union of 115.5 ± 41.4 days (range: 41-202 days). Persistent non-union was documented during follow-up in 26 patients; however, 10 of these did not complete later imaging surveillance, which precluded definitive determination of final union status. At least one postoperative complication occurred in 17 patients (37.8%). Hardware-related complications predominated, with screw loosening in 13 patients and painful screw malposition in one. Other complications comprised persistent pain (6.7%), wrist stiffness (4.4%), symptomatic non-union (6.7%), infection (2.2%), and progression to scaphoid non-union advanced collapse (SNAC) wrist (2.2%). Reoperation was performed in 16 patients (35.6%; 95% confidence interval (CI): 23.2-50.2%), most commonly for screw removal (n = 13). Three patients went on to have vascularised bone grafting, and one underwent wound washout with concurrent screw removal for infection. Among 15 patients with a documented reoperation date, the mean time to reoperation was 404.3 days (median: 308 days; IQR: 207.5-448.5 days).

Conclusions

Percutaneous bone grafting and screw fixation for scaphoid non-union was associated with a substantial complication burden and a high reoperation rate. Although minimally invasive techniques offer theoretical biological advantages, careful patient selection is essential. Further comparative studies are required to establish the role of this technique relative to established open reconstructive methods.

## Introduction

Scaphoid non-union continues to be a major challenge in wrist surgery. Failure of union may lead to progressive carpal collapse, degenerative arthritis, chronic pain, and functional impairment [1]. Open reduction and internal fixation with bone grafting remains the most widely accepted treatment strategy, with union rates consistently reported between 80% and 95% [2-4].

Percutaneous fixation techniques were developed to reduce surgical morbidity by preserving soft tissues and vascular supply [5,6]. Available minimally invasive approaches include percutaneous screw fixation alone [7], arthroscopic-assisted fixation [8], arthroscopic-assisted bone grafting with internal fixation [9,10], and techniques that incorporate distal radius decompression. More recently, percutaneous bone grafting and screw fixation have been introduced to enhance the biological environment while retaining the advantages of a minimally invasive approach [11,12]. Despite increasing interest, the current evidence on minimally invasive fixation of scaphoid non-union remains limited and is largely based on retrospective case series, with substantial heterogeneity in patient selection, surgical technique, and outcome reporting.

Across these studies, union rates are reported to be consistently high, generally exceeding 80%, and short- to mid-term functional outcomes are described as satisfactory. However, no clear advantage of one minimally invasive technique over another has been demonstrated. Recent systematic reviews have likewise found comparable union rates and functional outcomes between arthroscopic and open reconstruction techniques, while noting the low quality of the available evidence and the absence of comparative trials [13,14].

More importantly, the literature remains focused primarily on union and short-term functional outcomes. Reporting on the incidence of subsequent surgical procedures is notably lacking. Complications and reoperations are reported inconsistently and often incompletely, making it difficult to determine accurately the true risk profile of minimally invasive management. This study aimed to evaluate clinical outcomes, complication rates, and reoperation rates following percutaneous bone grafting and screw fixation for scaphoid non-union in a large cohort over 10 years.

## Materials and methods

Surgical technique

A retrograde guidewire was inserted through a small volar stab incision along the longitudinal axis of the scaphoid under image intensifier guidance. The scaphoid was then cannulated and drilled using the Acutrak drill system. Cancellous bone graft was harvested from the iliac crest using a disposable trephine biopsy needle. After appropriate trimming, the graft was advanced over the guidewire and impacted into the non-union site. The guidewire was then repositioned centrally within the scaphoid, and definitive fixation was achieved with an Acutrak headless compression screw. The final position of the screw and reduction of the non-union were confirmed under image intensifier in multiple planes and found to be satisfactory (Figure 1). Postoperatively, the wound was protected with a crepe bandage for two weeks, with gentle wrist use advised for six weeks.

**Figure 1:**
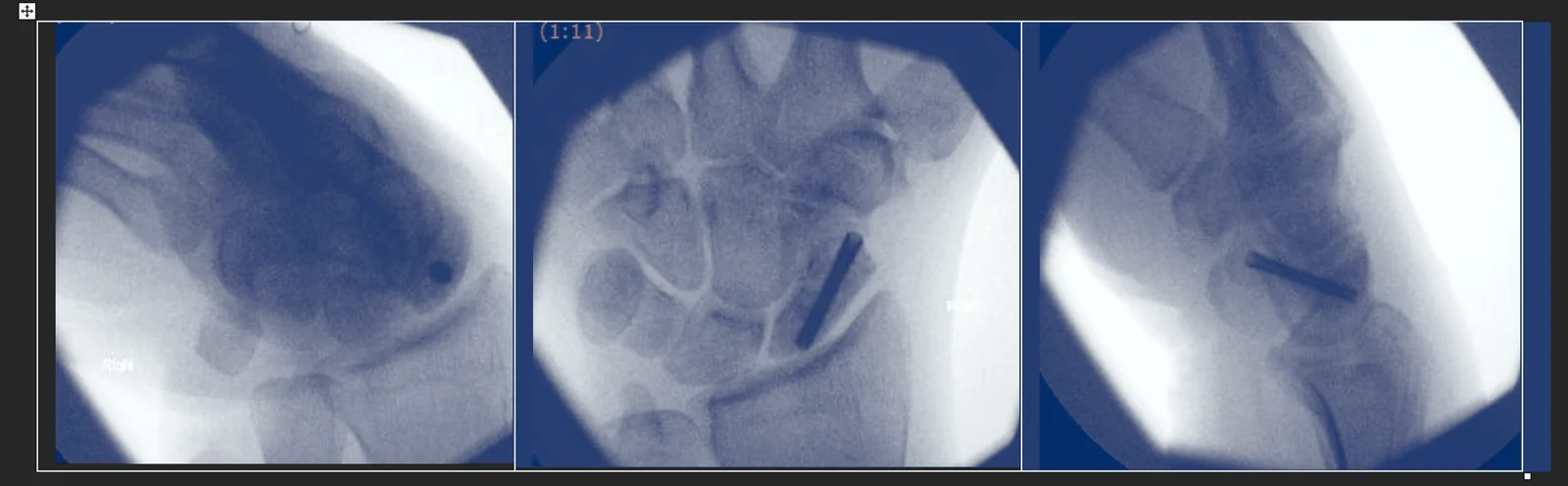
Intraoperative images of screw position after fixation This is an actual image taken while performing the procedure in one of the cases included in the study

Study details

Following institutional approval, a retrospective single-centre study of consecutive patients who had undergone percutaneous iliac crest bone grafting combined with headless compression screw fixation for scaphoid non-union between January 2010 and December 2020 was conducted. The study was carried out at Darlington Memorial Hospital, located in the North East of England. Patients were identified from a prospectively maintained operative database. Clinical records, operative reports, and radiographic images were reviewed retrospectively.

Demographic and clinical data collected included age, sex, indication for surgery, location of the scaphoid non-union, date of surgery, radiographic union, postoperative complications, subsequent surgical procedures, date of the last outpatient follow-up, and date of any revision procedure. The date of last follow-up was defined as the most recent documented clinical review before discharge from follow-up, a recorded non-attendance (DNA), or the last available clinical assessment. Data were analysed in Microsoft Excel (Microsoft Corp., Redmond, WA), and descriptive statistics were used to summarise the study population and outcomes.

Inclusion and exclusion criteria

Patients were eligible for inclusion if they had an established scaphoid non-union, defined as failure of fracture healing for more than six months, and had been treated with percutaneous iliac crest bone grafting and headless compression screw fixation. Only patients without a humpback deformity were included. Patients with acute scaphoid fractures or a humpback deformity requiring open corrective surgery were excluded.

Outcome measures

The primary outcome measures were radiographic union and the need for reoperation. The secondary outcome was postoperative complications. Radiographic union was assessed by the treating surgeon during routine outpatient follow-up and was independently reviewed by the study investigator. Union was defined radiographically as the presence of bridging trabeculae across the original non-union site on standard plain radiographs. Where plain radiographs were inconclusive, a CT scan was performed to confirm osseous union. Reoperations were identified by reviewing outpatient clinic records, operative records, and the institutional Picture Archiving and Communication System (PACS), including subsequent radiographic examinations where available.

Statistical analysis

Statistical analysis was carried out in Microsoft Excel (Microsoft Corp.). Continuous variables were summarised as the mean and standard deviation (SD) or the median and interquartile range (IQR), as appropriate. Categorical variables were summarised as frequencies and percentages. The overall reoperation rate was calculated as the number of patients who underwent an actual subsequent surgical procedure divided by the total number of patients in the study. The 95% confidence interval (CI) for the reoperation proportion was calculated using the Wilson method. Because only 16 reoperation events occurred in the study cohort, multivariable modelling was considered to carry a risk of overfitting. The analysis was therefore primarily descriptive and exploratory. As this was a retrospective cohort study, the sample size was determined by the number of consecutive eligible patients available during the predefined study period, rather than by an a priori prospective power calculation. Forty-five patients met the inclusion criteria and were included in the analysis. The relatively small sample size and number of reoperation events were considered when interpreting statistical associations and are acknowledged as limitations of the study.

## Results

The study included 45 consecutive patients with accessible clinical records. The mean age was 26.29 ± 11.15 years (range: 16-63 years). The majority of patients were male (41/45, 91%). Most non-unions involved the scaphoid waist (41/45, 91%), with the remaining four cases involving proximal pole non-unions. Four patients either did not attend their early postoperative follow-up or attended only once following surgery (Table 1).

**Table 1:** Summary of demographic characteristics, clinical outcomes, complications, and reoperations DNA: did not attend; SNAC: scaphoid non-union advanced collapse; CI: confidence interval; IQR: interquartile range

Variable	Values
Age	26.29 ± 11.15 years (range: 16–63)
Sex	41 (91%) males; 4 (9%) females
Indication	41/45 (91%) scaphoid waist non-unions
	4/45 (9%) proximal pole non-unions
Mean time to union	115.5 ± 41.4 days (range: 41–202 days)
	16.5 ± 6 weeks (range: 6–28 weeks)
Union status	15 confirmed unions
	26 non-unions
	10 did not complete follow-up (DNA)
	16 confirmed non-unions
Complications	13 loose screw and associated pain; 1 eccentric screw; 2 wrist stiffness; 1 infection; 1 progression to SNAC
Reoperation/second surgery	16 (35.6%) patients (95% CI: 23.2%–50.2%)
	13 screw removals
	3 vascularised bone grafts
	1 washout and screw removal
Reoperation/third surgery	(4 patients) 2/4 wrist denervation, ¼ vascularised bone graft, ¼ proximal scaphoid excision
		Period to reoperation	404.3 days (57 weeks) (range: 9–1,771 days)
(Median: 308 days; IQR: 207.5–448.5 days)		
Mean follow-up duration	Mean: 591.9 ± 508.1 days
	Median: 502 days (IQR: 216–653.5 days)

Follow-up duration was determined as the interval from the date of surgery to the date of the last documented follow-up. Among the 45 cases, 43 had a recorded final follow-up and were included in the follow-up duration analysis, while two patients who were recorded as DNA were excluded from this calculation. The mean follow-up duration was 591.9 ± 508.1 days, with a median of 502 days (IQR: 216-653.5 days), which corresponds to a mean follow-up of approximately 19.4 ± 16.7 months and a median of 16.5 months (IQR: 7.1-21.5 months).

Radiographic union

Radiographic union was confirmed in 15 patients. The mean time to first documented union was 115.5 ± 41.4 days (range: 41-202 days). Twenty-six patients were documented as having persistent non-union during follow-up. However, 10 of these patients did not complete subsequent imaging surveillance, as they did not attend further follow-up; therefore, their final union status could not be definitively established (Figure 2).

**Figure 2:**
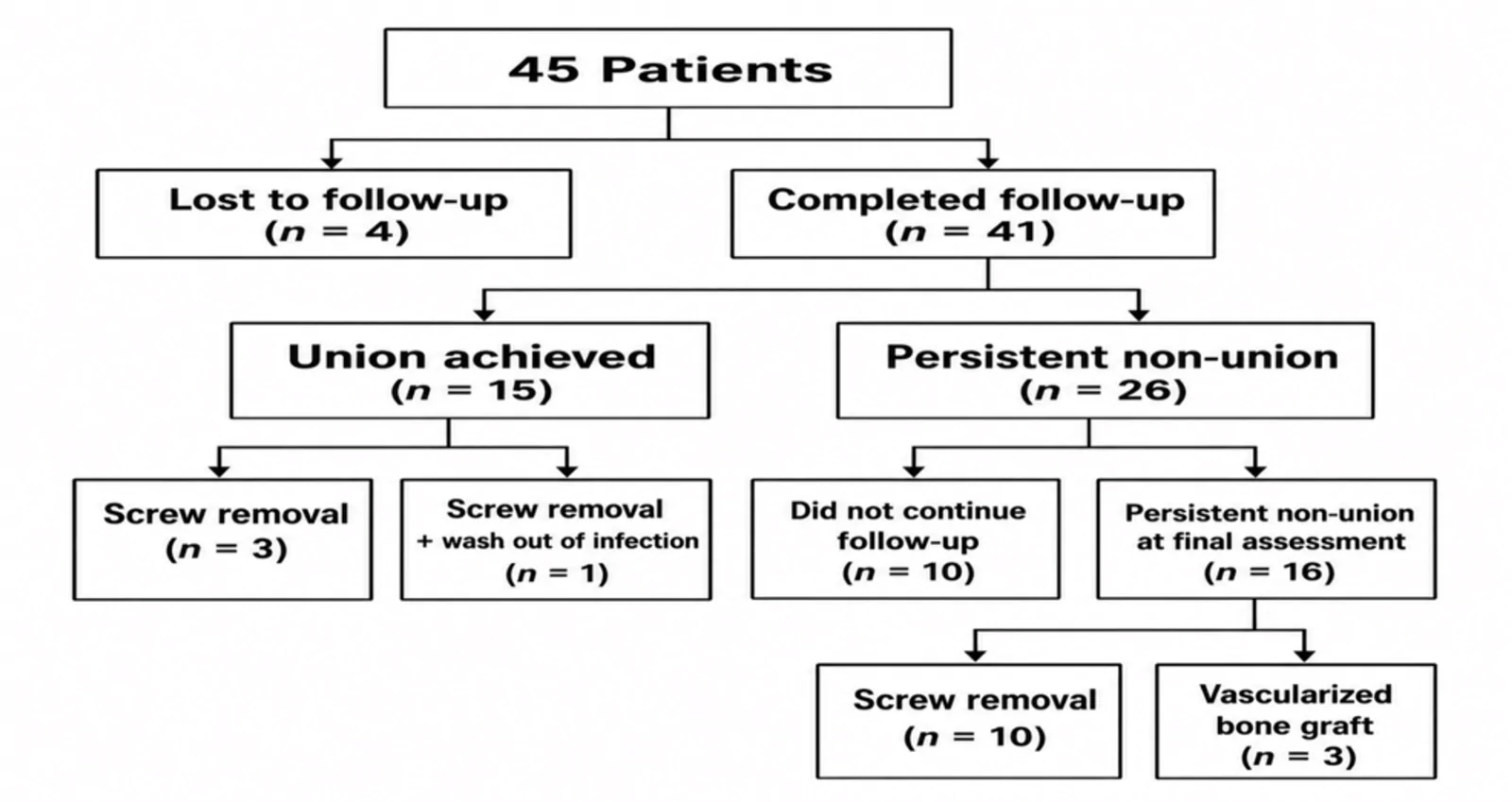
Flow diagram of follow-up and clinical outcomes following percutaneous scaphoid bone grafting and screw fixation

Complications

Seventeen of 45 patients (37.8%) experienced at least one postoperative complication. Hardware-related complications accounted for the majority of adverse events, comprising 13 cases of screw loosening and one case of screw malposition associated with pain. Other reported complications included persistent pain in three patients (6.7%), wrist stiffness in two (4.4%), symptomatic non-union in three (6.7%), infection in one (2.2%), and progression to scaphoid non-union advanced collapse (SNAC) wrist in one patient (2.2%). Some patients experienced more than one complication, and therefore individual complication categories are not mutually exclusive.

Reoperations

Sixteen of 45 patients (35.6%) underwent subsequent surgery, corresponding to a reoperation rate of 35.6% (95% CI: 23.2%-50.2%; Wilson score method). The most common secondary procedure was screw removal (n = 13). Three patients subsequently underwent vascularised bone grafting, while one patient underwent wound washout for infection with concurrent screw removal (Figure 2). Among the 15 patients with a documented date of reoperation, the mean time to reoperation was 404.3 days, with a median of 308 days (IQR: 207.5-448.5 days; range: 9-1,771 days). One patient was excluded from the time-to-reoperation analysis because the definitive date of reoperation was unavailable, as the procedure was performed outside the treating trust.

Of the 16 patients who underwent a second operation, four subsequently required a third operation. The indications and procedures comprised wrist denervation in two patients, vascularised bone grafting in one patient, and excision of the proximal scaphoid in one patient.

## Discussion

The principal finding of this study was the unexpectedly high rate of hardware-related complications and secondary surgery following percutaneous bone grafting and screw fixation for scaphoid non-union. The most notable reason for secondary surgery was metalware removal following loosening, which likely reflects both the technical and biological challenges of percutaneous fixation in scaphoid non-union. Unlike acute fractures, established scaphoid non-unions frequently demonstrate sclerosis, cystic degeneration, bone resorption, fracture gapping, humpback deformity, and altered scaphoid morphology.

These pathological changes reduce bone stock, compromise screw purchase, and limit the ability to achieve stable interfragmentary compression, often necessitating debridement and bone grafting to restore scaphoid anatomy and achieve union [5]. Furthermore, it is possible that percutaneous bone grafting may alter screw trajectory and seating within the drilled channel, thereby creating uneven force distribution and microscopic voids around the implant. In the scaphoid, where the bone is largely covered by hyaline cartilage with limited soft-tissue containment, these voids may allow synovial fluid to enter and promote peri-implant bone resorption. Previous biomechanical studies have demonstrated that micromotion and fluid pressure at the bone-implant interface can impair osseointegration, promote fibrous tissue formation, and contribute to progressive implant loosening [15-18].

Although percutaneous approaches are attractive due to reduced surgical exposure and preservation of vascularity, the current results suggest that these theoretical advantages may not translate into predictable clinical success in all patients. Hardware complications occurred in over one-third of cases, and one in three patients required further surgery.

The present series compares unfavourably with published outcomes following traditional open reconstruction for scaphoid non-union. Munk and Larsen [19] reported union rates of 80-91% across more than 5,000 cases, while a recent meta-analysis of 7,671 patients by Duncumb et al. [20] demonstrated overall union rates approaching 89%, rising to 96.5% in studies excluding proximal pole fractures and avascular necrosis. Similarly, Merrell et al. [2] reported a 94% union rate following screw fixation combined with bone grafting. In contrast, the confirmed union rate in the current series was substantially lower, although interpretation is limited by incomplete follow-up. This study showed that the majority of cases involved young male adults, and younger age and male gender have been identified as risk factors for loss to follow-up in several studies [22].

Although several small cohort studies have reported union rates ranging from 70% to 95% following percutaneous screw fixation for scaphoid non-union [6,7], there remains a paucity of literature describing the combined use of percutaneous bone grafting and screw fixation and the associated reoperation rate over prolonged timescales. Sener et al. [10] is one of the few available series to utilise this technique; however, the study included only 11 patients and demonstrated an 82% union rate. Their procedure was identical to the current study, except that the thumb was splinted in a cast for four weeks postoperatively [6]. Ohta et al. [11] reported full union in six cases of non-union treated with percutaneous iliac bone graft and headless screw fixation; however, their procedure included an additional step [7]. After drilling a hole for the screw, curettage of the cystic lesion was performed using a small sharp curette passed through the distal hole. Postoperatively, the thumb was splinted in a thumb spica for six weeks. Both available reports were limited in terms of the number of cases and the reporting of long-term outcomes. Likewise, Capo et al. (2012) [22] reported an 11/12 (92%) success rate for union using percutaneous fixation of scaphoid non-union. However, in a relatively larger cohort, Munsch et al. [23] reported a union rate of 71% in 21 patients following percutaneous screw fixation without bone grafting for scaphoid non-union, highlighting the potential limitations of percutaneous fixation in chronic non-union cases. The current study, by contrast, reflects the realistic outcome of this procedure, as it incorporates a larger number of cases (45) and reports subsequent procedures required.

Published reports [24] of percutaneous fixation have generally demonstrated favourable outcomes; however, most studies have involved highly selected patient populations and relatively small cohorts. In contrast, the present study reflects routine clinical practice over a prolonged period and may therefore provide a more representative assessment of the limitations of this technique.

The study has several limitations. Its retrospective design introduces potential bias, including observer bias in radiographic assessment. The absence of standardised functional outcome measures, variability in follow-up duration, and lack of multivariable analysis limit assessment of patient-centred outcomes and predictors of complications or reoperation. Most importantly, incomplete radiographic follow-up and the small sample size preclude accurate determination of the true union rate and represent significant limitations when interpreting efficacy. Nevertheless, this series highlights a complication profile that has received limited attention in the literature and suggests that caution should be exercised when considering percutaneous management of established scaphoid non-union.

## Conclusions

Percutaneous iliac crest bone grafting combined with headless compression screw fixation for scaphoid non-union was associated with a 38% complication rate and a 35% reoperation rate in this series. Hardware-related complications were common and frequently necessitated secondary intervention. Although minimally invasive techniques remain appealing, these findings suggest that outcomes may be less predictable than those reported for traditional open reconstruction.
